# MicroRNA-29b-3p promotes intestinal permeability in IBS-D via targeting TRAF3 to regulate the NF-κB-MLCK signaling pathway

**DOI:** 10.1371/journal.pone.0287597

**Published:** 2023-07-10

**Authors:** Yongfu Wang, Wei Ke, Jianfeng Gan, He Zhu, Xiangyu Xie, Guodong He, Shan Liu, Yusheng Huang, Hongmei Tang

**Affiliations:** 1 Department of Traditional Chinese Medicine, The First Affiliated Hospital of Zhengzhou University, Zhengzhou, Henan, China; 2 The First Clinical Medical College of Guangzhou University of Chinese Medicine, Guangzhou, China; 3 The First Affiliated Hospital, Guangzhou University of Chinese Medicine, Guangzhou, Guangdong, China; 4 Lingnan Medical Research Center, Guangzhou University of Chinese Medicine, Guangzhou, Guangdong, China; 5 The Second Affiliated Hospital, Guangzhou University of Chinese Medicine, Guangzhou, Guangdong, China; Guangdong Medical University, CHINA

## Abstract

Irritable bowel syndrome with predominant diarrhea (IBS-D) is characterized by increased intestinal permeability. Previous studies have shown that the microRNA-29 gene is involved in the regulation of intestinal permeability in patients with IBS-D. NF-κB was proved to play a key role in inflammatory response of intestine and resultant disruption of tight junction integrity, whose activity could be inhibited by TNF Receptor-Associated Factor 3 (TRAF3). However, the exact mechanism that induces increased intestinal permeability in IBS-D patients has not been clarified. In this study, we found that microRNA-29b‑3p (miR-29b-3p) was significantly upregulated, while TRAF3 was decreased and the NF-κB-MLCK pathway was activated within the colonic tissue of IBS-D patients. Subsequently, we confirmed the targeting relationship between miR-29b-3p and TRAF3 through a double-luciferase reporter assay. Lentivirus transfection of NCM460 cells with miR-29b-3p-overexpressing and -silencing vectors demonstrated that the expression of TRAF3 was negatively correlated with the level of miR-29b-3p. The NF-κB/MLCK pathway was activated in the miR-29b-3p-overexpressing group and inhibited to some extent in the miR-29b-3p-silencing group. Results in WT and miR-29 knockout mice showed that miR-29b-3p levels were increased, TRAF3 levels were decreased, and the NF-κB/MLCK signaling was activated in the WT IBS-D group as compared with the WT control group. The protein levels of TRAF3 and TJs in the miR-29b^-/-^ IBS-D group were partially recovered and NF-κB/MLCK pathway indicators were, to a certain extent, decreased as compared with the WT IBS-D group. These results suggested that miR-29b-3p deletion enhances the TRAF3 level in IBS-D mice and alleviates the high intestinal permeability. In brief, through the analysis of intestinal tissue samples from IBS-D patients and miR-29b^-/-^ IBS-D mice, we showed that miR-29b-3p is involved in the pathogenesis of intestinal hyperpermeability in IBS-D via targeting TRAF3 to regulate the NF-κB-MLCK signaling pathway.

## 1. Introduction

Irritable bowel syndrome (IBS) is a prevalent gastrointestinal disorder characterized by abdominal pain associated with defecation or altered bowel habits. Irritable bowel syndrome with predominant diarrhea (IBS-D) is one of the major subtype of IBS [1], which imposes a substantial burden on patients and the healthcare system, resulting in decreased quality of life for patients [2]. At present, the pathogenesis of IBS-D is not fully understood, which may involve genetic predisposition, intestinal hyperpermeability, altered intestinal barrier function, immune dysregulation, and brain-gut imbalance [3, 4]. Therefore, it is crucial to explore the underlying pathomechanism of IBS-D in order to develop effective treatments for this disease.

Intestinal hyperpermeability proved to play an important part in the pathogenesis of IBS-D [5]. Maintenance of intestinal permeability relies on an intact intestinal barrier function. The intimate connections between cytoskeletal structures and intercellular tight junctions (TJs) contribute to the integrity of the intestinal barrier [6]. Cytoplasmic claudins, occludin, and junctional adhesion molecules (JAMs) proteins are important TJs proteins that determine intestinal permeability [7]. JAMs play a crucial role in forming cell polarity and cell junctions [8]. Claudin-1 is involved with certain channel and sealing functions [9]. Occludin is an essential component of the maintenance and regulation of the intestinal barrier function [10]. All these are important intestinal epithelial TJs, once destructed, can lead to increased gut permeability, which could finally induce local or systemic inflammation [11]. Interestingly, and vice versa, increased permeability of the colonic epithelium could also be a consequence of inflammatory response and disruption of TJ integrity, through which process, NF-κB was shown to play a key role via multiple cellular signaling pathways [12]. Indeed, NF-κB is extensively involved in numerous inflammatory diseases, including inflammation in the gastrointestinal tract [13]. The most abundant form of NF-κB activated by pathological stimulation through a typical pathway is the p50:p65 heterodimer [14]. And furtherly, myosin light-chain kinase (MLCK) is an important signaling node that regulates the integrity of TJs. Previous research has demonstrated that TNF-α activates NF-κB via a pathway involving MLCK, thereby influencing the intestinal barrier function [15].

MicroRNAs are short, non-coding RNA molecules that regulate the expression of target genes by complementary pairing with the target gene mRNA 3 UTR [16]. Studies have reported that microRNA-29 is involved in regulating IBS-D intestinal epithelial barrier function [17]. The increased expression of miR-29 in the intestinal tissues of IBS-D patients resulted in the decreased expression of NKRF [18], a transcriptional silencer of NF-κB [19]. Moreover, TRAF3 can inhibit the activity of NF-κB by continuously mediating the degradation of NF-κB-induced kinase [20]. In the previous study, it was found that the expression level of miR-29b-3p was increased and the expression level of TRAF3 was decreased in the intestinal tissues of IBS-D patients, and there was a targeted inhibition relationship between miR-29b-3p and TRAF3. However, the mechanism of regulation of IBS-D by miR-29b-3p through targeted inhibition of TRAF3 has not been reported. In order to explore the specific role of miR-29b-3p in IBS-D, this study was verified by clinical tissue samples, animal experiments and cell experiments. This study aims to elucidate the mechanism by which miR-29b-3p regulates the NF-κB-MLCK signaling pathway involved in IBS-D intestinal hyperpermeability through targeted inhibition of TRAF3. This will provide a new idea for the establishment of biomarkers for clinical diagnosis of IBS-D.

## 2. Materials and methods

### 2.1. Patients

Subjects enrolled in this study included 10 IBS-D patients and 10 healthy controls (June 1, 2017 to December 31, 2017). Diagnosis of IBS-D was according to Rome IV criteria [21]. Table 1 describes the clinical parameters of these subjects. Two days before colonoscopy, 5 mL of fasting antecubital venous blood was obtained from the subjects. Colonic mucosa samples were collected during the colonoscopy at the First Affiliated Hospital of Guangzhou University of Chinese Medicine (Guangzhou, China). The clinical research project was approved by the Medical Research Ethics Committee of the First Affiliated Hospital of Guangzhou University of Chinese Medicine (No. ZYYECKYJ-2017-011). All subjects signed the informed consent. The study protocol conformed to the standards set by the Declaration of Helsinki. Authors have access to information that identifies individual participants both during and after data collection.

**Table 1:** Demographics and clinical characteristics of patients with diarrhea-predominant irritable bowel syndrome and healthy controls.

Features	IBS-D patients	Controls	P value
n	10	10	NA
Age in year	31.4±6.82	31.9±6.92	0.873
Gender, male: Female	6:4	6:4	1
Duration of disease in year	2.95 (1.50, 4.00)	NA	NA
Defecation frequency	3.00 (2.50, 3.60)	1.00 (0.80, 1.00)	0.000
BSFS score	5.50 (5.00, 6.00)	4.00 (3.75, 4.00)	0.000
IBS-SSS	217.50 (175.00, 275.00)	NA	NA

The data are presented as the mean ± SD or the median (Q1, Q3). BSFS: Bristol stool form scale; IBS-D: Irritable bowel syndrome with predominant diarrhea; IBS-SSS: Irritable bowel syndrome symptom severity scale; NA: Not applicable.

### 2.2. Animals and IBS-D model establishment

Heterozygous miR-29b^+/-^ mice were purchased from the Shanghai Model Organisms Center , Inc. (SCXK 2014–0002, Shanghai, China) and bred to obtain miR-29b^-/-^ mice (miR-29b knockout mice). CRISPR/Cas9 technology was used to cause functional loss of the *miR-29b* gene by introducing mutations through non-homologous recombination repair. The process was manipulated according to the previous protocol of our laboratory [22]. In brief, Cas9 mRNA and sgRNA were obtained by in vitro transcription, and then microinjected into the zygotes of C57BL/6J mice to obtain F0 generation mice. The homozygous miR-29b-deficient mice were screened by PCR, and then they were paired with C57BL/6J mice for amplification and reproduction. The experimental mice were housed in standard laboratory conditions with a temperature of 20–25°C, relative humidity of 50 ± 5%, and a 12 h light-dark cycle. The genotype of the miR-29b^-/-^ mice and wild type (WT) C57BL/6 mice were identified by PCR using the following primers: mmu-miR-29b Forward: AGGGGGCAGGGTCTCATTAGCA and mmu-miR-29b Reverse: CCCACCCCCTTCCCCTCAG.

Twenty-four male WT and miR-29b^-/-^ mice were randomly assigned into four groups: 1) WT control, 2) WT IBS-D, 3) miR-29b^-/-^ control, and 4) miR-29b^-/-^ IBS-D groups (n = 6). In order to establish the IBS-D model, WT IBS-D and miR-29b^-/-^ IBS-D groups were given an intracolonic administration of acetic acid once (3%, diluted in distilled water) at the age of 6 weeks. The water avoidance stress (WAS) test was conducted the next day. Briefly, mice were placed on a small platform (10 × 8 × 8 cm) in a basin (45 × 25 × 25 cm) with warm water 1 cm below the platform for 1 h every day for 10 consecutive days. At the end of the study, mice were anesthetized by inhalation of isoflurane and sacrificed by cervical decapsulation. All animal experiments were conducted in accordance with the National Institutes of Health guide for the care and use of Laboratory animals and approved by the Institutional Animal Care and Welfare Committee of Guangzhou University of Chinese Medicine (TCMF1-2019001).

### 2.3. Assessment of visceral sensitivity and fecal water content in mice

After the model was established, the visceral sensitivity of the mice was assessed by calculating the abdominal withdrawal reflex (AWR) as previously reported [23]. The AWR score (0–4) was used to grade pain responses at different degrees. Two blinded researchers recorded the AWR scores at each pressure.

The next day after the modeling manipulation was finished all groups of mice were kept in separate metabolic cages for 60 minutes. The feces of mice in each cage were collected and weighed. After the feces were completely dried in oven at 60°C for 48 h, they were weighed again, and the moisture content of the feces was calculated as follows: water content (%) = (wet weight of the feces − dry weight of the feces) / wet weight of the feces × 100%.

### 2.4. Histological observation

Colonic tissues of clinical subjects and mice were fixed in 4% paraformaldehyde (Biosharp, Hefei, China), embedded with paraffin, and cut into 5-mm slices. Slices were stained with hematoxylin and eosin (H&E, Yeasen, Shanghai, China) and imaged for analysis. The images were evaluated and scored according to the protocols listed in Table 2 [24, 25].

**Table 2:** The scoring scale used for histology.

Score	Severity of inflammation	Crypt damage	Ulceration
0	Rare inflammatory cells in the lamina propria	Intact crypt	0 foci of ulceration
1	Increased numbers of granulocytes in the lamina propria	Intact crypt	0 foci of ulceration
2	Confluent inflammatory cells extended to submucosa	Loss of basal 1/3 of crypt	1–2 foci of ulceration
3	Moderate inflammatory cell infiltrates	Loss of basal 2/3 of crypt	1–2 foci of ulceration
4	Marked inflammatory cell infiltrates	Loss of entire crypt	3–4 foci of ulceration
5	Marked transmural inflammation	Change in epithelial surface caused by erosion	Confluent or extensive ulceration

### 2.5. Detection of serum D-LA (D-lactic acid), DAO (Diamine oxidase), and LPS (Lipopolysaccharide) levels

Levels of serum D-LA, DAO, and LPS in humans and mice were detected using ELISA kits by following the manufacturer’s protocols (D-LA ELISA kit, No. JL13858; DAO ELISA kit, No. JL11855; LPS ELISA kit, No. JL20691). Briefly, blood samples were centrifuged at 4°C, 3000 rpm, 4500 g for 15 min, and supernatant was collected for detection. Each blood sample was tested twice to obtain an average OD value at 450 nm by a microplate reader (ThermoFisher Scientific, Waltham, MA, USA). The above kits were purchased from Shanghai Jianglai Biotechnology Co., LTD (Shanghai, China).

### 2.6. Cell culture and transfection

NCM460 cells, a normal human colon mucosal epithelial cell line (Shanghai Yaji Biotechnology Co., LTD, Shanghai, China), were maintained in Dulbecco’s modified eagle medium (Gibco, California, USA) containing 100 U/ml penicillin, 100 μg/ml streptomycin (Gibco, California, USA) at 37°C in a 5% CO_2_ humidified atmosphere. Before transfection, the resuscitated NCM460 cells were seeded into 24-well plates and cultured to 40% confluence. Then, the cells were transfected with 1 × 10^7^ TU (transducing units) / ml virus of miR-29b-3p UP, miR-29b-3p inhibition, miR-29b-3p UP negative control, and miR-29b-3p inhibition negative control (Shanghai Genechem Co., Ltd, Shanghai, China) following the instructions. Total RNA and protein were extracted after 72 h of cell culture for further use.

### 2.7. Reverse transcription quantitative PCR (RT-qPCR)

The colon tissues and NCM460 cells were collected, and total RNA was extracted using the TRIzol reagent (Invitrogen Life Technologies, Carlsbad, CA, USA). The expression levels of the miRs and mRNAs were determined via RT-qPCR. The purified RNA was synthesized to cDNA by a first-strand synthesis kit (Takara, Tokyo, Japan) according to the instructions of manufacturer. RT-qPCR was performed on a Bio-Rad CFX96 Touch Real-Time PCR Detection System using the amplification conditions reported previously [26]. The primer sequences used in the experiment were synthesized by Sangon Biotech Co., Ltd. (Shanghai, China) (Table 3). The relative miRNA and mRNA expression levels were calculated using the 2^−ΔΔCT^ formula and normalized to *U6* and *GAPDH* [22].

**Table 3:** RT-qPCR primer sequences used in this study.

Gene	Forward primer (5’ to 3’)	Reverse primer (3’ to 5’)
miR-29b-3p (Human)	CGCGCGCGTAGCACCATTTGAAATCAG	AACGCTTCACGAATTTGCGT
miR-29b-3p (Mouse)	CGCGCGCGTAGCACCATTTGAAATCA	AACGCTTCACGAATTTGCGT
U6 (Human, Mouse)	CTCGCTTCGGCAGCACA	AACGCTTCACGAATTTGCGT
TRAF3 (Human)	CTGGCTCTTCAGATCTATTGTCGG	TCCCGGTATTTACACGCCTT
TRAF3 (Mouse)	AGATGCTCCGAAACAACGAGT	CCGCACTTTTGTCTACGCTCT
Tnf-alpha (Human)	GTGACAAGCCTGTAGCCCATGTT	TTATCTCTCAGCTCCACGCCATT
Tnf-alpha (Mouse)	ACTCCAGGCGGTGCCTATGT	GTGAGGGTCTGGGCCATAGAA
Nfkb1 (Human)	GCCTCCACAAGGCAGCAAATA	CACCACTGGTCAGAGACTCGGTAA
Nfkb1 (Mouse)	TCCGGGAGCCTCTAGTGAGAA	TCCATTTGTGACCAACTGAACGA
RELA (Human)	GACGCATTGCTGTGCCTTC	TTGATGGTGCTCAGGGATGAC
RELA (Mouse)	ATTGCTGTGCCTACCCGAAAC	TTTGAGATCTGCCCTGATGGTAA
MLCK (Human)	GCGGATTTCCTCATGCAGG	AGCACATGCTTTGGTTTTCCT
MLCK (Mouse)	AAGGGAGCTGACCTAACCCAG	TTGCTACGGACAATTCCAGGG
GAPDH (Human)	GCACCGTCAAGGCTGAGAAC	TGGTGAAGACGCCAGTGGA
GAPDH (Mouse)	TGTGTCCGTCGTGGATCTGA	TTGCTGTTGAAGTCGCAGGAG

### 2.8. Western blot analysis

Total and phosphorylated proteins were isolated in RIPA lysis buffer (Beyotime, Shanghai, China) with protease inhibitors and phosphatase inhibitors as previously described [26]. The protein concentration was measured using a Bicinchoninic Acid protein assay kit (Thermo Fisher Scientific, Waltham, MA, USA). The extracted proteins were then separated via sodium dodecyl sulfate-polyacrylamide gel electrophoresis and transferred to polyvinylidene difluoride membranes (Thermo Fisher, Shanghai, China). Subsequently, membranes were incubated at 4°C for 12 h with the primary antibodies (Table 4). A horseradish peroxidase-conjugated secondary antibody was incubated with the membranes at 25°C for 2 h. The relative expression of the proteins was quantified and standardized to the expression of GAPDH by using Quantity One 4.6.2 Software (Bio-Rad, California, USA).

**Table 4:** Antibody information used in this study.

Antibody Name	Dilution Concentration	Cat Number	Company
TRAF3 (Human, Mouse)	1:1000	AF5380	Affinity Biosciences
TNF-α (Human, Mouse)	1:1000	AF7014	Affinity Biosciences
p-p65 (Human, Mouse)	1:1000	AF2006	Affinity Biosciences
p65 (Human, Mouse)	1:1000	AF5006	Affinity Biosciences
MLCK (Human, Mouse)	1:1000	AF5314	Affinity Biosciences
p-MLC (Human, Mouse)	1:1000	AF8618	Affinity Biosciences
MLC (Human, Mouse)	1:1000	AF5423	Affinity Biosciences
Claudin-1 (Human, Mouse)	1:1000	ab180158	Abcam
Occludin (Human, Mouse)	1:1000	ab21632	Abcam
JAM-A (Human, Mouse)	1:1000	ab180821	Abcam
GAPDH (Human, Mouse)	1:2000	AF7021	Affinity Biosciences
Secondary antibody	1:3000	S0010	Affinity Biosciences

### 2.9. Dual-luciferase reporter assay

For TRAF3 luciferase reporter construction, 400-bp fragments of the TRAF3 3’ UTR, 5’ UTR, or TRAF3-coding region containing the predicted binding site were inserted into the pmir-GLO reporter vector (Tsingke Biotechnology Co., Ltd., Beijing, China) to generate the 3’ UTR, 5’ UTR, or TRAF3-coding region WT luciferase reporters. The binding site mutant vector was then generated using the Site Directed Mutagenesis kit (Tsingke Biotechnology Co., Ltd., Beijing, China). For the assay, NCM460 cells were co-transfected with miR-29b-3p mimic and TRAF3-WT, miR-29b-3p mimic negative control (NC) and TRAF3-WT, miR-29b-3p mimic and TRAF3-MUT, or miR-29b-3p mimic NC and TRAF3-MUT in 24-well plates by Lipofectamine 3000 (Thermo Fisher, Massachusetts, USA). After incubating for 24 h, the cells were harvested. Firefly luciferase activities were measured with a Luciferase Assay System (Promega, Beijing, China) and normalized to Renilla luciferase activity, according to the manufacturer’s protocol.

### 2.10. Statistical analysis

Statistical analysis was performed using SPSS software (version 23.0, SPSS Inc., Chicago, USA). All values are expressed as the mean ± standard deviation (S.D.). Differences between two groups were analyzed by a Student’s *t* test, and an one-way ANOVA was used to analyze differences among multiple groups. *P* < 0.05 were considered to be statistically significant.

## 3. Results

### 3.1. Subject demographic and clinical characteristics

Demographic and clinical characteristics included IBS-D patients and healthy volunteers as shown in Table 1. Subjects enrolled in this study included 10 IBS-D patients and 10 healthy volunteers, with a male to female ratio of 6:4. The mean age of IBS-D patients was 31.40, while that of the healthy control group was 31.90, which was not statistically different. The BSFS scale showed that the median score of IBS-D patients was 5.50 and that of the control group was 4.00, indicating a significant statistical difference (*P* < 0.01). The IBS Symptom Severity Scale [27] showed that patients with IBS tended to score moderate.

### 3.2 Mild intestinal inflammation and hyperpermeability in IBS-D patients

**Fig 1:**
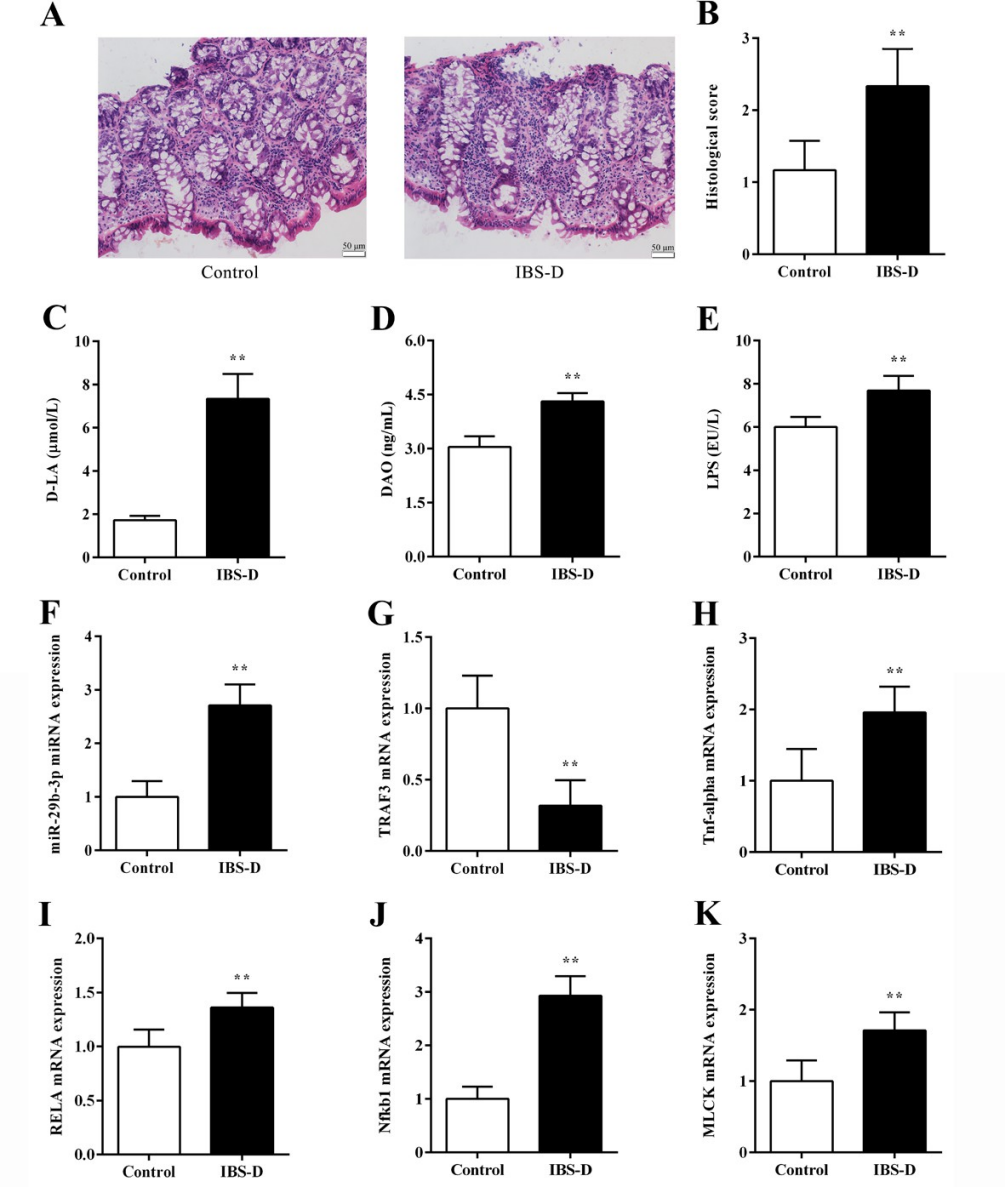
Evaluation of colon tissue integrity, intestinal permeability indexes, expression of miR-29b-3p and its potential target genes in IBS-D patients and healthy controls. (A) Representative H&E images of the colon (magnification ×200). (B) Histological score. (C) Serum D-LA levels in humans. (D) Serum DAO levels in humans. (E) Serum LPS levels in humans. (F) Relative *miR-29b-3p* miRNA levels in the colons of humans. Relative mRNA levels of (G) *TRAF3*, (H) *Tnf-alpha*, (I) *RELA*, (J) *Nfkb1* and (K) *MLCK* in the colons of humans. Data are shown as the mean ± S.D. (n = 6). ^**^ *P* < 0.01 vs. healthy controls.

As shown in Fig 1A and 1B, histological examination revealed neither apparent infiltration of inflammatory cells nor crypt destruction in the healthy control group, whereas the IBS-D group showed partial loss of goblet cells, disappearance of crypt epithelium, and mild infiltration of inflammatory cells. Colon histopathological scores showed significant differences between IBS-D patients and healthy controls. Additionally, compared with healthy controls, IBS-D patients had significantly increased intestinal permeability indices [28], as indicated by serum levels of D-LA, DAO, and LPS (*P*_all_ < 0.01, Fig 1C–1E), suggesting that there was mild inflammation and high permeability in the colon of patients with IBS-D.

### 3.3. Expression of miR-29b-3p and its potential target gene in IBS-D patients

We detected the expression of miR-29b-3p in the colonic mucosa specimen of clinical subjects by RT-qPCR and found that the level of miR-29b-3p in IBS-D patients was significantly increased as compared to that of healthy controls (*P* < 0.01) (Fig 1F). In order to uncover the mechanism underlying the pathogenesis of miR-29b-3p in IBS-D, we measured the expression level of TRAF3 and its potential downstream genes. The results showed that the level of TRAF3 was significantly downregulated in the colonic mucosa of the IBS-D patients group as compared to the control group (*P* < 0.01, Fig 1G). On the contrary, the mRNA expression levels of Tnf-alpha, RELA, Nfkb1, and MLCK were significantly upregulated in IBS-D patients as compared to the controls (*P*_all_ < 0.01, Fig 1H–1K). These results suggested that increased colonic permeability in IBS-D patients may be related to the regulation of miR-29b-3p and TRAF3.

### 3.4. MiR-29b-3p targets TRAF3 in NCM460 cells

**Fig 2:**
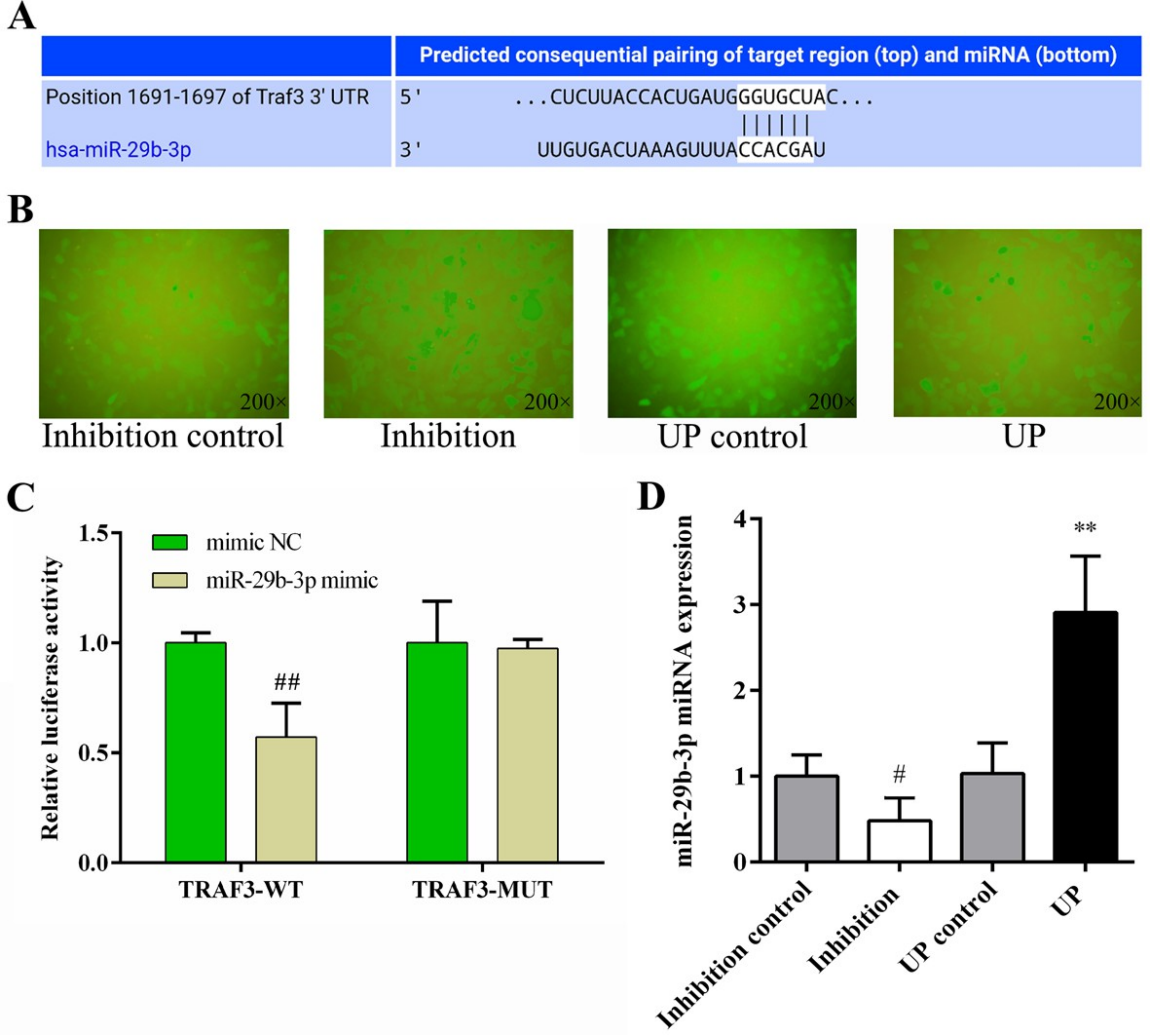
The targeting relationship between *miR-29b-3p* and *TRAF3* was verified, and the efficiency of lentivirus-mediated *miR-29b-3p* transfection in NCM460 cells was evaluated. (A) The 3’ UTR binding site of *miR-29b-3p* to *TRAF3*. (B) The overexpression or inhibition of *miR-29b-3p* in NCM460 cells was identified by fluorescent markers. (C) Dual luciferase reporter gene detection of *miR-29b-3p* and *TRAF3*. (D) The overexpression or inhibition of miR-29b-3p in NCM460 cells was verified by RT-qPCR. Data are shown as the mean ± S.D. (n = 3). ^##^ *P* < 0.01 vs. mimic NC group.

It has been predicted that TRAF3 is the direct target of miR-29b-3p and that the target relationship is evolutionarily conserved [29, 30]. To confirm this, we performed online miRNA target analysis (http://starbase.sysu.edu.cn) and dual-luciferase reporter gene tests on miR-29b-3p and TRAF3 genes. Online analysis and screening of database unveiled the binding site between miR- 29-3p and the TRAF3 3’ UTR (Fig 2A). The results of dual luciferase reporter gene tests showed that mimic NC and miR-29b-3p mimic had no effect on the mutant vector and no-load activity of the target gene. The miR-29b-3p mimic significantly inhibited the activity of the wild-type TRAF3 vector (*P* < 0.01). After site-specific mutation, the miR-29b-3p mimic had no inhibitory effect on the mutant TRAF3 vector (Fig 2C). These results confirmed that miR-29b-3p could bind directly to the 3’ UTR of TRAF3 mRNA.

### 3.5. Efficiency of miR-29b-3p transfection into NCM460 cells by lentivirus

NCM460 cells successfully transfected expressed green fluorescent protein (GFP). 72 hours post-transfection, NCM460 cells were observed by fluorescence microscope and photographed, and it was found that the fluorescence ratio of each group was more than 90%. The results showed that virus expressing miR-29b-3p up, miR-29b-3p inhibition, miR-29b-3p up control, and miR-29b-3p inhibition control were successfully transfected into NCM460 cells and stably expressed (Fig 2B). RT-qPCR was used to verify the expression levels of miR-29b-3p in each group, and it was found that compared with the Inhibition control group, transfection of the miR-29b-3p inhibitor significantly inhibited the expression of miR-29b-3p in NCM460 cells (*P* < 0.05). Compared with the up control group, transfection with miR-29b-3p mimic significantly increased the expression level of miR-29b-3p in NCM460 cells (*P* < 0.01, Fig 2D). These results showed that miR-29b-3p and its control group were successfully transferred into NCM460 cells and expressed stably.

### 3.6. Expression of miR-29b-3p target gene TRAF3 and NF-κB/MLCK pathway-related indicators in NCM460 cells after transfection

**Fig 3:**
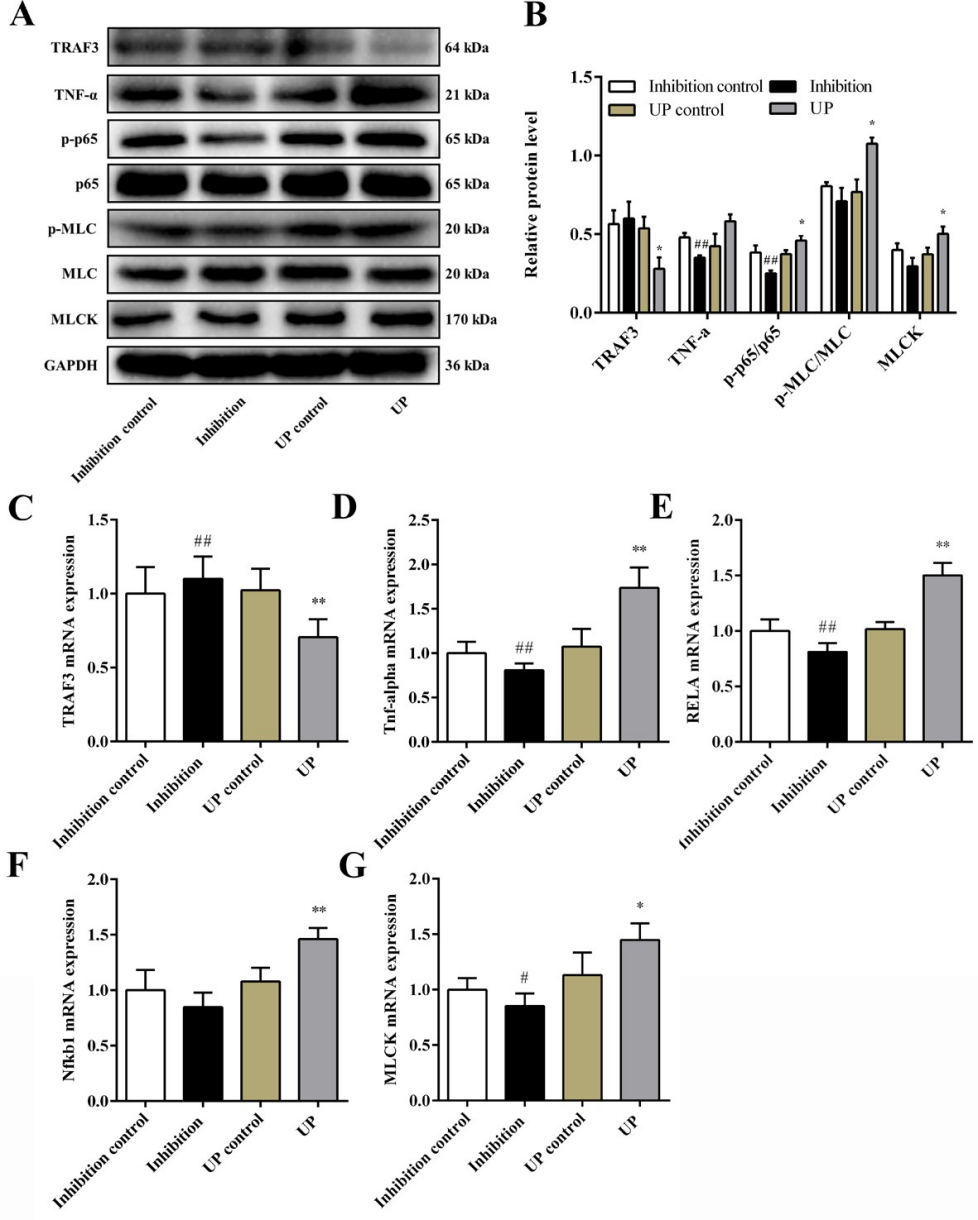
The expression of the genes and proteins in the TRAF3 and NF-κB/MLCK pathway in NCM460 cells after transfection. (A) TRAF3 and NF-κB/MLCK pathway representative western blot images. (B) Relative protein expression of TRAF3, TNF-α, p-p65/p65, p-MLC/MLC, and MLCK. NCM460 cells mRNA levels of (C) *TRAF3*, (D) *Tnf-alpha*, (E) *RELA*, (F) *Nfkb1*, and (G) *MLCK*. Data are shown as mean ± the S.D. (n = 3–6). ^#^ *P* < 0.05, ^##^ *P* < 0.01 vs. Inhibition control group; ^*^ *P* < 0.05, ^**^ *P* < 0.01 vs. UP control group.

In order to evaluate whether miR-29b-3p could effectively regulate TRAF3 expression in vitro, we further examined TRAF3 expression after miR-29b-3p mimic or inhibitor transfection in NCM460 cells. Both TRAF3 protein and mRNA expression were decreased after transfection of miR-29b-3p mimic in NCM460 cells (*P* < 0.05 or *P* < 0.01). Conversely, TRAF3 protein and mRNA levels were increased in NCM460 cells treated with the miR-29b-3p inhibitor (Fig 3A–3C). The experimental results showed that TRAF3 expression was inversely associated with miR-29b-3p levels.

**Fig 4:**
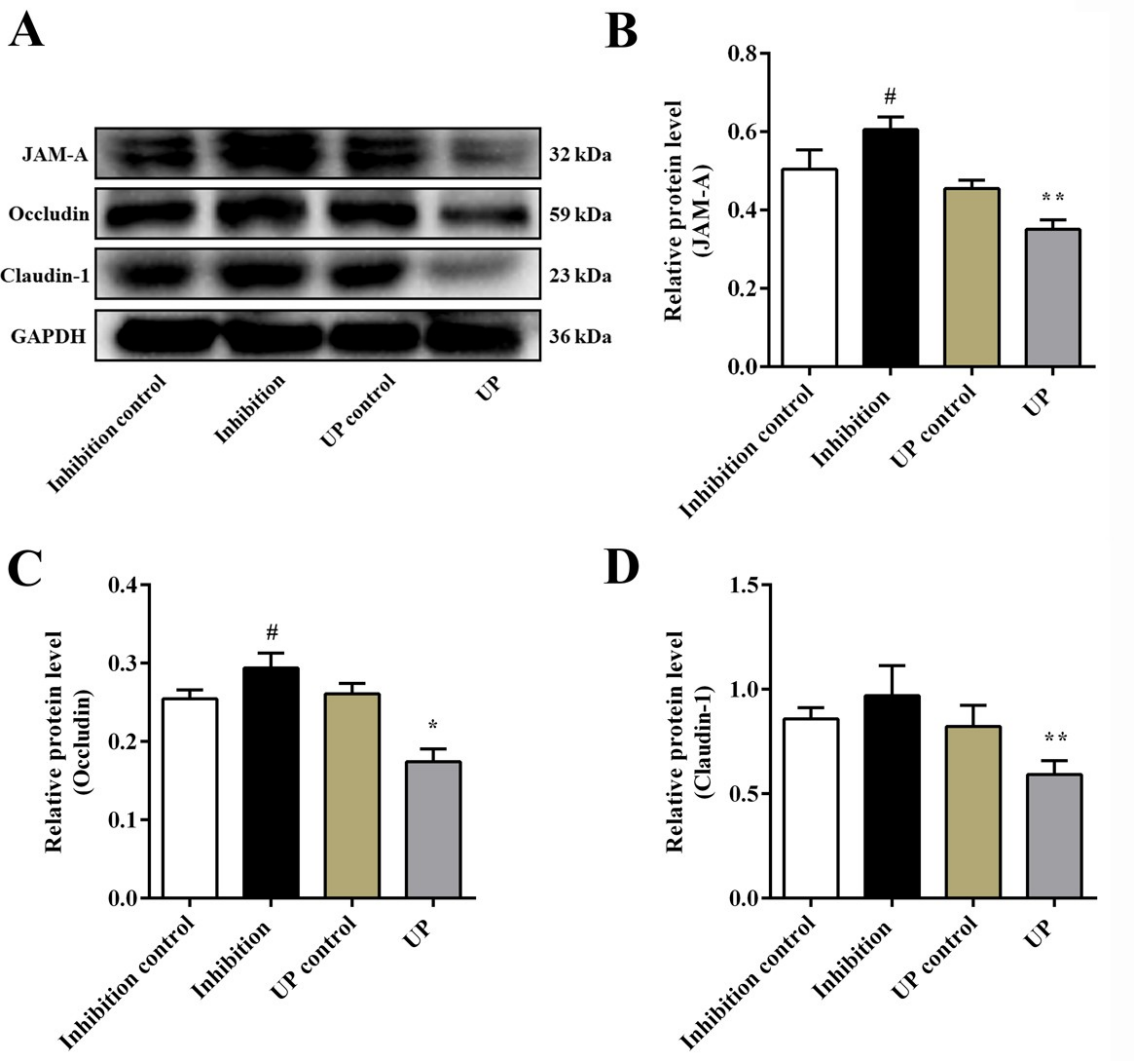
Expression of TJ proteins in NCM460 cells after transfection. (A) Representative western blot images of TJ proteins. Relative protein expression of (B) JAM-A, (C) Occludin, and (D) Claudin-1. Data are shown as mean ± the S.D. (n = 3). ^#^ *P* < 0.05 vs. inhibition control group; ^*^ *P* < 0.05, ^**^ *P* < 0.01 vs. UP control group.

Accordingly, we also detected the expression of NF-κB/MLCK pathway-related indicators in NCM460 cells after transfection with a miR-29b-3p mimic or inhibitor. The protein expression of TNF-α, p-p65/p65, p-MLC/ MLC, and MLCK in the UP group was significantly increased as compared to the UP control group (*P*_all_ < 0.05). In contrast, the expression of these proteins in the inhibition group were decreased as compared to the inhibition control group (Fig 3A and 3B; TNF-α, p-p65/p65, *P* < 0.01). Similarly, the mRNA levels of Tnf-alpha, RELA, Nfkb1, and MLCK were all dramatically elevated in the UP group as compared with the UP control group (*P* < 0.05 or *P* < 0.01), whereas the inhibition group decreased the mRNA levels of these indicators as compared to the inhibition control group (Fig 3D–3G; Tnf-alpha, RELA, *P* < 0.01; MLCK, *P* < 0.05). Moreover, as shown in Fig 4A–4D, the protein levels of JAM-A, occluding, and claudin-1 in the UP group were remarkably decreased as compared to the UP control group (JAM-A, Claudin-1, *P* < 0.01; Occludin, *P* < 0.05), whereas the expression of JAM-A, Occludin, and Claudin-1 were increased in the inhibition group as compared with the inhibition control group (JAM-A, Occludin, *P* < 0.05). These results suggested that there is a negative feedback regulation between TRAF3 and the NF-κB/MLCK pathway at the cellular level. Overexpression of miR-29b-3p inhibits TRAF3 levels, resulting in activation of the NF-κB/MLCK pathway and destruction of TJs.

### 3.7. miR-29b^-/-^ mouse identification and IBS-D model evaluation

**Fig 5:**
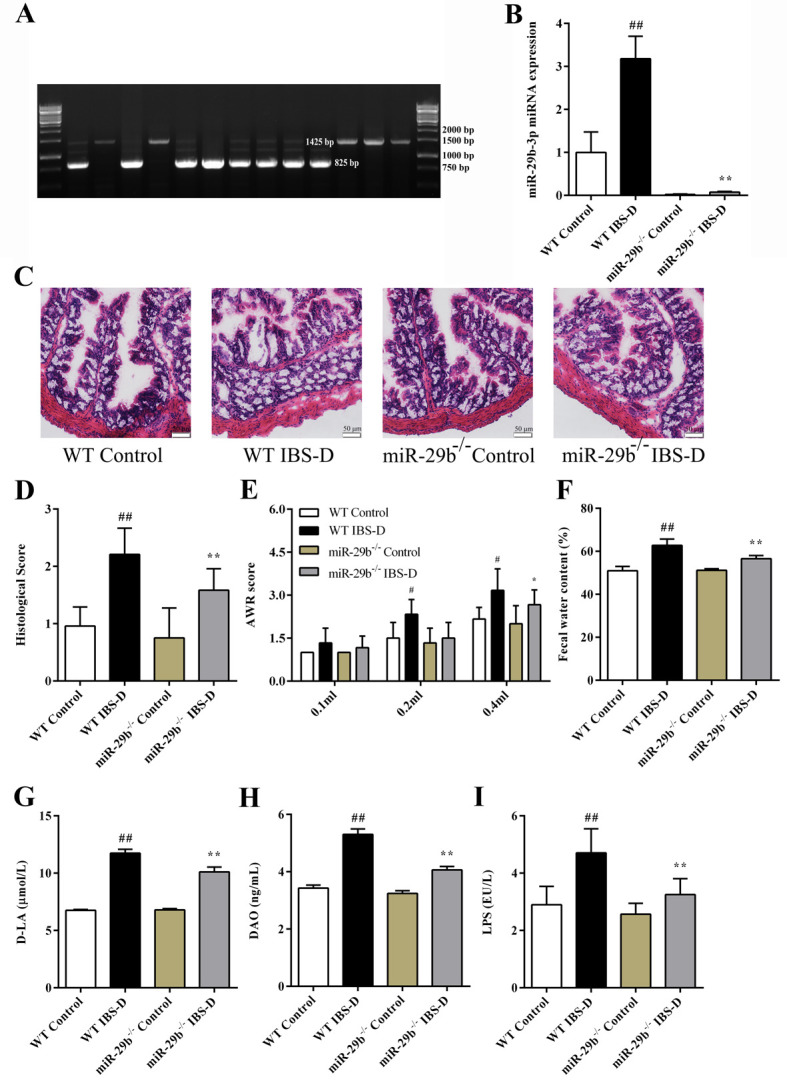
Verification of the genotype, colon tissue integrity, AWR score, fecal water content and intestinal permeability indexes in experimental mice. (A) Representative agarose gel electrophoresis image. (B) Relative *miR-29b-3p* miRNA levels in the colons of mice. (C) Representative H&E images of mice colons (magnification ×200). (D) Histological score of mice in each group. (E) The AWR score of mice in each group. (F) The fecal water content of mice in each group. (G) Serum D-LA levels in mice. (H) Serum DAO levels in mice. (I) Serum LPS levels in mice. Data are shown as the mean ± S.D. (n = 6). ^#^ *P* < 0.05, ^##^ *P* < 0.01 vs. WT Control group; ^*^ *P* < 0.05, ^**^ *P* < 0.01 vs. WT IBS-D group.

We next utilized miR-29b^-/-^ mice in order to verify the effect of miR-29b-3p on intestinal hyperpermeability during IBS-D. The genotype of the miR-29b^-/-^ mice was confirmed via PCR amplification and agarose gel electrophoresis by using DNA isolated from the tails of each mouse. According to the instructions, the wild-type mice had a band at 1425 bp, and the mutant band was 825 bp (Fig 5A). PCR identification results of miR-29b-3p are shown in Fig 5B. miR-29b-3p expression in WT IBS-D mice was significantly higher than that in WT control group, but miR-29b-3p expression in both miR-29b^-/-^ control group and miR-29b^-/-^ IBS-D group was maintained at a very low level.

The AWR score is a suitable indicator of visceral sensitivity of IBS-D animals [23], therefore, we tested AWR score of each mice. After IBS-D modeling, the AWR score (0.2 ml, 0.4 ml) of WT IBS-D mice was significantly elevated as compared to that in the WT control group (Fig 5E, *P*_all_ < 0.05), while the AWR score in the miR-29b^-/-^ IBS-D group was lower than that of the WT IBS-D group (AWR score 0.4 ml, *P* < 0.05). The results showed that miR-29b knockout reduced the intestinal sensitivity of IBS-D mice to a certain extent. The fecal water content of WT mice and miR-29b^-/-^ mice increased significantly after IBS-D modeling, but the increase in fecal water content was more obvious in the WT IBS-D group (Fig 5F, *P*_all_ < 0.01).

Intestinal permeability indices (D-LA, DAO, and LPS) in the WT IBS-D model group were also significantly increased as compared to those in the WT control group (*P*_all_ < 0.01, Fig 5G–5I). Histological examination of the colon tissue of the WT IBS-D group showed mild edema, impaired mucosal integrity, and inflammatory cell infiltration (Fig 5C and 5D). However, intestinal permeability indices and the colonic histological score of the miR-29b^-/-^ IBS-D group were alleviated as compared with WT IBS-D group (colon histological score, D-LA, DAO, and LPS *P*_all_ < 0.01). All the results together confirmed that the IBS-D mouse model was successfully established using intracolonic acetic acid stimulation and water stress, and the modeling effect was suitable. In IBS-D mice, knockdown of miR-29b reduced the high permeability of the intestinal epithelium to a certain extent and maintained the structural integrity of the colonic epithelium.

### 3.8. Expression of miR-29b-3p target gene TRAF3 and NF-κB/MLCK pathway in miR-29b^-/-^ mice

**Fig 6:**
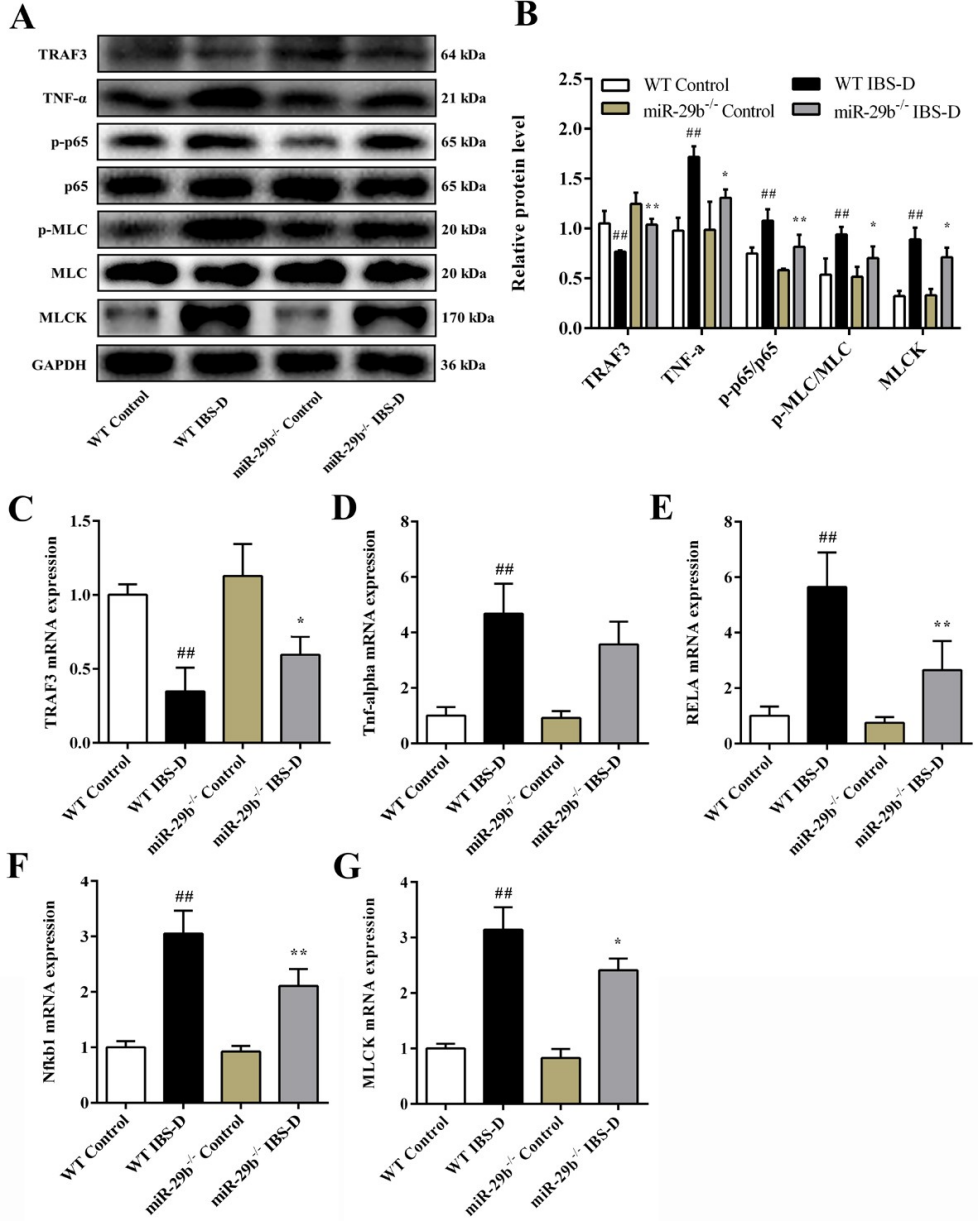
Expression of the genes and proteins in the TRAF3 and NF-κB/MLCK pathway targets in the colon tissues of mice. (A) TRAF3 and NF-κB/MLCK pathway representative western blot images. (B) Relative protein expression of TRAF3, TNF-α, p-p65/p65, p-MLC/MLC, and MLCK. Relative mRNA levels of (C) *TRAF3*, (D) *Tnf-alpha*, (E) *RELA*, (F) *Nfkb1*, and (G) *MLCK*. Data are shown as mean ± the S.D. (n = 3–6). ^#^ *P* < 0.05, ^##^ *P* < 0.01 vs. WT Control group; ^*^ *P* < 0.05, ^**^ *P* < 0.01 vs. WT IBS-D group.

In order to confirm that the TRAF3 signaling pathway regulates intestinal permeability in miR-29b^-/-^ mice, we detected the levels of TRAF3, TNF-α, p65, p50, MLCK, MLC, p-MLC, Claudin-1, Occludin, and JAM-A in the colonic tissues of animals. As illustrated in Fig 6A and 6B, the protein expression levels of TNF-α, p-p65/p65, p-MLC/MLC, and MLCK in the WT IBS-D group were increased while protein expression levels of TRAF3 was decreased as compared to WT control group (*P*_all_ < 0.01). However, the miR-29b^-/-^ IBS-D group had decreased expression of TNF-α, p-p65/p65, p-MLC/ MLC, MLCK and increased expression of TRAF3 as compared to the WT IBS-D group (*P* < 0.05 or *P* < 0.01). Similarly, compared with the control group of both WT and miR-29b^-/-^ mice, the mRNA levels of Tnf-alpha, RELA, Nfkb1, and MLCK in the IBS-D group were all significantly increased, while TRAF3 mRNA level were significantly decreased (*P*_all_ < 0.01). However, compared with WT IBS-D group, the mRNA levels of Tnf-alpha, RELA, Nfkb1, and MLCK were much lower while TRAF3 was much higher in miR-29b^-/-^ IBS-D group (*P* < 0.05 or *P* < 0.01, Fig 6C–6G).

**Fig 7:**
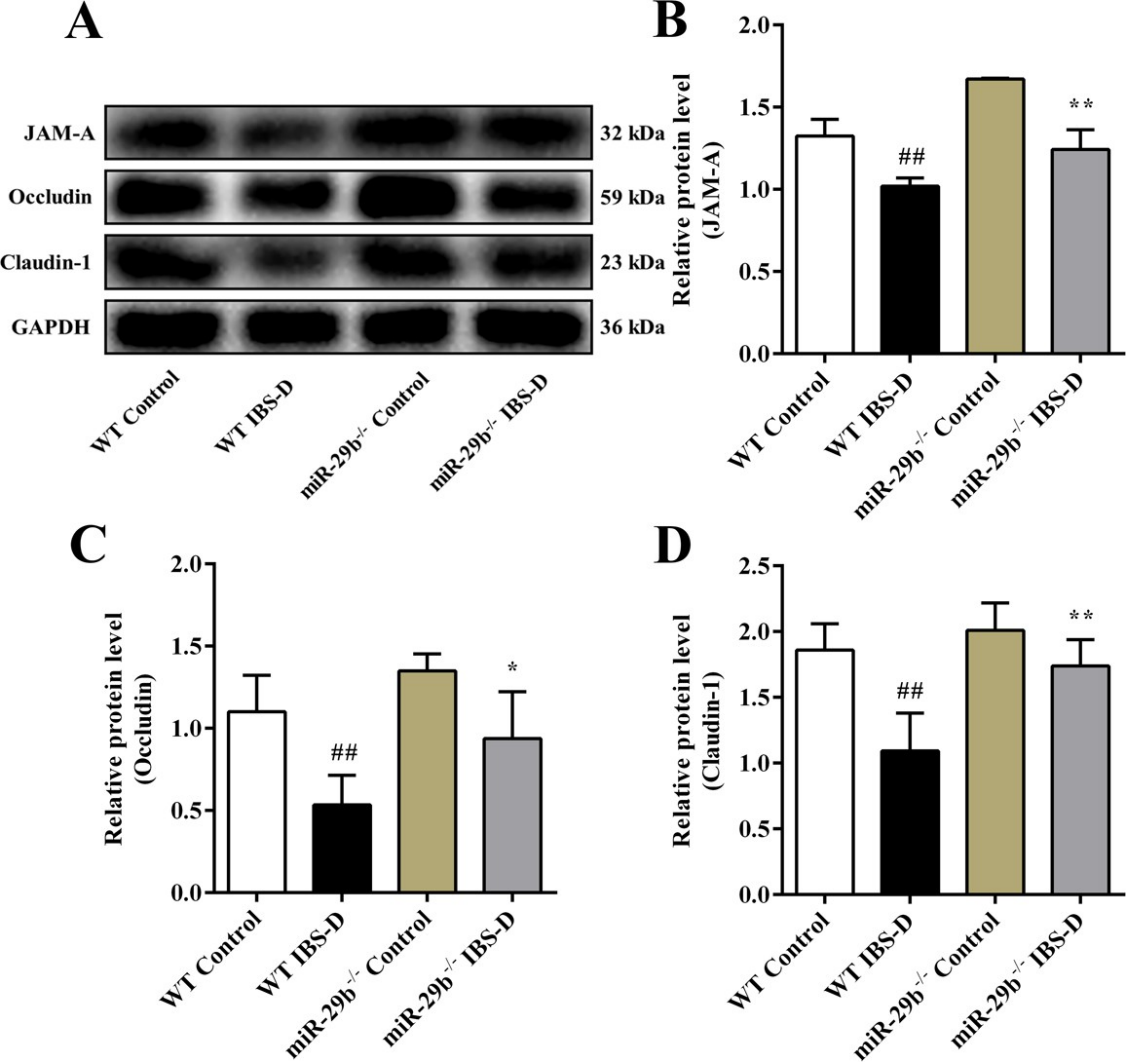
Expression of TJ proteins in the colon tissues of mice. (A) Representative western blot images of TJ proteins. Relative protein expression of (B) JAM-A, (C) Occludin and (D) Claudin-1. Data are shown as mean ± the S.D. (n = 3). ^##^ *P* < 0.01 vs. WT Control group; ^*^ *P* < 0.05, ^**^ *P* < 0.01 vs. WT IBS-D group.

The colonic protein expression of JAM-A, Occludin, and Claudin-1 were remarkably decreased in the WT IBS-D group as compared with the WT control group (*P*_all_ < 0.01, Fig 7A–7D). On the contrary, the expression level of TJ proteins in the miR-29b^-/-^ IBS-D group were partially restored as compared with the WT IBS-D group (*P* < 0.05 or *P* < 0.01). All these data suggested that miR-29b^-/-^ mice subjected to the IBS-D model had alleviated intestinal barrier function disruption and intestinal hyperpermeability as compared with WT IBS-D group via targeting TRAF3 and inhibiting the NF-κB/MLCK signaling pathway.

## 4. Discussion

IBS-D is a common gastrointestinal disorder in clinic characterized by recurrent abdominal pain and diarrhea, whose etiopathogenesis is associated with hyperpermeability and hypersensitivity of the intestinal tract [31]. Previous studies have shown that mild inflammation and disruption of the intestinal epithelial barrier play an crucial role in the progression of IBS-D, as both allow increased amounts of D-LA produced by the intestinal flora to enter the blood circulation through the damaged mucosal cells [32]. DAO is a catabolic pathway enzyme that frequently accumulates within the intestinal mucosa and microvilli. During ischemia or hypoxia, DAO can be released into the blood, and its serum concentration is positively correlated with the integrity of the intestinal mucosa [33]. Similarly, LPS has been shown to be closely related to intestinal barrier integrity resulting from dysregulated intestinal homeostasis [34]. Therefore, D-LA, DAO, and LPS are widely used to assess intestinal cell damage and dysfunction in IBS-D, and all these three indicators are negatively correlated with intestinal mucosal permeability. Our results demonstrated higher serum levels of D-LA, DAO, and LPS in IBS-D model mice than control mice, with miR-29b^-/-^ mice in much lower level compared with corresponding WT mice.

Intestinal hyperpermeability induced by acetic acid stimulation leads to damage of the intestinal mucosal barrier and allows for invasion of pathogenic bacteria [28]. What’s more, anxiety and depression are common in patients suffering from functional gastrointestinal disorders, such as IBS-D [35]. Repeated exposure to WAS successfully reproduced sustained visceral hyperalgesia after stress sensitization in animals in parallel with psychiatric comorbidity of IBS-D patients in clinic, which is widely accepted as an suitable IBS model [36]. Therefore, we established IBS-D mice model by combination of acetic acid enema and WAS. In the current study, H&E staining of intestinal biopsies from IBS-D patients and IBS-D mice exhibited mild inflammatory cell infiltration, crypt damage, and TJ destruction. Serum D-LA, DAO, and LPS levels were also remarkably increased (*P*_all_ < 0.01) in IBS-D patients and IBS-D mice. In addition, the visceral sensitivity of IBS-D model mice was enhanced as indicated by AWR score and the severity of diarrhea in IBS-D mice was aggravated as indicated by fecal water content, which were consistent with the abdominal pain and pathological diarrhea manifestations of IBS-D patients in clinic.

MicroRNAs, which are small RNAs capable of regulating protein levels, have attracted interest in recent years as diagnostic biomarkers, pathogenic indicators, and potential therapeutic targets. Members of the miR-29b family include miR-29b-3p, and miR-29b-5p, and their mature sequences have high similarity. Nonetheless, due to the different isomers, their determined functions differ [37]. Recent studies have shown that miR-29b-3p plays an important role in the progress of IBS-D concerning intestinal permeability [18]. Studies have reported that miR-29b-3p plays a key role in ischemia-reperfusion injury by targeting TRAF3 [30]. Our dual-luciferase reporter gene assay confirmed that microRNA-29b-3p could target to TRAF3 in NCM460 cells. TRAF3 is an important signal transducer involved in the interferon regulator NF-κB and apoptotic signaling pathways [38], which is widely expressed in the brain, lung, heart, spleen, and liver and is a target gene of miR-29b-3p. A previous study reported an important β-cell-macrophage pathway mediated by miRNA-29-TRAF3 [39], while another study found that miR-29b-3p affects the triple-negative breast cancer cell line MDA-MB-231 by targeting TRAF3 and activating the NF-κB signaling pathway [40]. More importantly, studies revealed that microRNA-29 and its target genes NF-κB inhibitor (NKFR) and Claudin-1 play an important role in the pathogenesis of IBS-D as key regulators of intestinal permeability [18]. TNF-α, a common inflammatory cytokine, can promote the activation of the NF-κB pathway, which leads to an increase in intestinal permeability [41]. After activation of NF-κB, both p50 and p65 were significantly upregulated [42]. Studies have shown that NF-κB within the intestinal epithelium directly targets to MLCK and modulates the activity of MLCK. Specifically, activated NF-κB released p50 and p65 to act on MLCK [43]. Consequently, MLCK mediates the phosphorylation of MLC, causing contraction of the cytoskeleton of intestinal epithelial cells, eventually leading to TJ dysregulation and intestinal hyperpermeability [44].

In the current study, we found that the expression level of miR-29b-3p within the colonic mucosa of IBS-D patients was significantly increased, the expression level of its target gene TRAF3 was reversely decreased, and the related NF-κB/MLCK pathway indicators (TNF-α, p65, p50, and MLCK) were increased. These results suggested that miR-29b-3p and its target gene TRAF3 may be involved in the pathogenesis of IBS-D. To verify this hypothesis, we silenced and overexpressed miR-29b-3p in NCM460 cells and further assessed the function of miR-29b in miR-29b knockout mice.

In the cell experiment, we found that the expression of TRAF3 in NCM460 cells in the miR-29b-3p-overexpressing group was significantly reduced, which further verified the negative targeting relationship between miR-29b-3p and TRAF3. The elevation of miR-29b-3p significantly inhibited the normal expression of TRAF3. Due to the absence of TRAF3, the NF-κB pathway was activated, which resulted in the subsequent activation of TNF-α, NF-κB heterodimer p50/p65, and increased MLCK gene activity and protein expression. Phosphorylation of MLC proved to eventually lead to the disruption of TJs and increased permeability [45]. However, in the miR-29b-3p-silencing group, the expression of the miR-29b-3p gene was relatively decreased, resulting in enhanced expression of TRAF3, which inhibited the NF-κB/MLCK pathway to a certain extent. Our results confirmed a negative feedback regulation between TRAF3 and NF-κB/MLCK pathway at the cellular level. Overexpression of miR-29b-3p inhibited the level of TRAF3, which resulted in the activation of the NF-κB/MLCK pathway and disruption of TJs.

Furtherly, we compared the gene and protein expression of TRAF3, NF-κB/MLCK pathway-related indicators, and TJs in the colon tissue of IBS-D mouse model. It was found that compared with the WT control group, the expression of TRAF3 in the WT IBS-D group was significantly reduced, the NF-κB/MLCK pathway was activated, and the expression of TJs were significantly downregulated. Our experimental results confirmed that the expression levels of JAM-A, Occludin, and Claudin-1 were reduced in IBS-D mice, which was in parallel with the significantly increased intestinal permeability as indicated by enhanced serum levels of D-LA, DAO, and LPS in IBS-D model group. However, compared with WT IBS-D mice, miR-29b^-/-^ IBS-D mice showed less protein damage to JAM-A, Occludin, and Claudin-1 and alleviated intestinal hyperpermeability. These data suggested that miR-29b deficiency (or downregulation) could improve intestinal barrier function by targeting TRAF3 to inhibit the NF-κB/MLCK signaling pathway.

## 5. Conclusion

In summary, IBS-D patients and IBS-D model mice demonstrated increased miR-29b-3p expression, accompanied by reduced TRAF3 expression and activation of the NF-κB-MLCK signaling pathway, which downregulated the level of JAM-A, Claudin-1 and Occludin TJ proteins, resulting in damage to the intestinal epithelial barrier and resultant intestinal hyperpermeability. While in miR-29b knockout mice and miR-29b-3p-silenced NCM460 cells, activation of NF-κB-MLCK pathway was inhibited and the TJs and intestinal epithelial barrier function were restored. Hence, we speculated that the colon hyperpermeability of IBS-D patients may be regulated by the endogenous interaction between miR-29b-3p and TRAF3 along with its downstream NF-κB-MLCK pathway.

## Supporting information

S1 File (DOCX)

S1 Raw images (PDF)

## References

[1] Lovell RM, Ford AC. Global Prevalence of and Risk Factors for Irritable Bowel Syndrome: A Meta-analysis. Clin Gastroenterol Hepatol. 2012;10: 712–721. doi: 10.1016/j.cgh.2012.02.029 22426087

[2] Oka P, Parr H, Barberio B, Black CJ, Savarino E V., Ford AC. Global prevalence of irritable bowel syndrome according to Rome III or IV criteria: a systematic review and meta-analysis. Lancet Gastroenterol Hepatol. 2020;5: 908–917. 32702295 10.1016/S2468-1253(20)30217-X

[3] Ford AC, Sperber AD, Corsetti M, Camilleri M. Series Functional Gastrointestinal Disorders 2 Irritable bowel syndrome. Lancet. 2020;6736: 1–14. 10.1016/S0140-6736(20)31548-8 33049223

[4] Holtmann GJ, Ford AC, Talley NJ. Pathophysiology of irritable bowel syndrome. Lancet Gastroenterol Hepatol. 2016;1: 133–146. doi: 10.1016/S2468-1253(16)30023-1 28404070

[5] Hanning N, Edwinson AL, Ceuleers H, Peters SA, De Man JG, Hassett LC, et al. Intestinal barrier dysfunction in irritable bowel syndrome: a systematic review. Therap Adv Gastroenterol. 2021;14: 1–31. doi: 10.1177/1756284821993586 33717210 PMC7925957

[6] Ye X, Sun M. AGR2 ameliorates tumor necrosis factor-α-induced epithelial barrier dysfunction via suppression of NF-κB p65-mediatedMLCK/p-MLC pathway activation. Int J Mol Med. 2017;39: 1206–1214. 28339048 10.3892/ijmm.2017.2928 PMC5403182

[7] Van Itallie CM, Anderson JM. Claudins and epithelial paracellular transport. Annu Rev Physiol. 2006;68: 403–429. doi: 10.1146/annurev.physiol.68.040104.131404 16460278

[8] Steinbacher T, Kummer D, Ebnet K. Junctional adhesion molecule-A: functional diversity through molecular promiscuity. Cell Mol Life Sci. 2018;75: 1393–1409. doi: 10.1007/s00018-017-2729-0 29238845 PMC11105642

[9] Bertrand J, Ghouzali I, Guérin C, Bôle-feysot C, Gouteux M, Déchelotte P, et al. Glutamine Restores Tight Junction Protein Claudin-1 Expression in Colonic Mucosa of Patients With Diarrhea-Predominant Irritable Bowel Syndrome. 2015. 10.1177/0148607115587330 25972430

[10] Heinemann U. Structural Features of Tight-Junction Proteins. 2019; 1–24. 10.3390/ijms20236020 PMC6928914 31795346

[11] D’Antongiovanni V, Pellegrini C, Fornai M, Colucci R, Blandizzi C, Antonioli L, et al. Intestinal epithelial barrier and neuromuscular compartment in health and disease. World J Gastroenterol. 2020;26: 1564–1579. doi: 10.3748/wjg.v26.i14.1564 32327906 PMC7167418

[12] Li W, Gao M, Han T. Lycium barbarum polysaccharides ameliorate intestinal barrier dysfunction and inflammation through the MLCK-MLC signaling pathway in Caco-2 cells. Food Funct. 2020;11: 3741–3748. doi: 10.1039/d0fo00030b 32314770

[13] Taniguchi K, Karin M. NF-B, inflammation, immunity and cancer: Coming of age. Nat Rev Immunol. 2018;18: 309–324. doi: 10.1038/nri.2017.142 29379212

[14] Giridharan S, Srinivasan M. Mechanisms of NF-κB p65 and strategies for therapeutic manipulation. J Inflamm Res. 2018;11: 407–419. 30464573 10.2147/JIR.S140188 PMC6217131

[15] Jin Y, Blikslager AT. The regulation of intestinal mucosal barrier by myosin light chain Kinase/Rho kinases. Int J Mol Sci. 2020;21: 15–17. doi: 10.3390/ijms21103550 32443411 PMC7278945

[16] Treiber T, Treiber N, Meister G. Regulation of microRNA biogenesis and its crosstalk with other cellular pathways. Nat Rev Mol Cell Biol. 2019;20: 5–20. doi: 10.1038/s41580-018-0059-1 30728477

[17] Guo D, Yang J, Ling F, Tu L, Li J, Chen Y, et al. Elemental Diet Enriched with Amino Acids Alleviates Mucosal Inflammatory Response and Prevents Colonic Epithelial Barrier Dysfunction in Mice with DSS-Induced Chronic Colitis. J Immunol Res. 2020;2020. doi: 10.1155/2020/9430763 32855978 PMC7443247

[18] Zhou Q, Costinean S, Croce CM, Brasier AR, Merwat S, Larson SA, et al. MicroRNA 29 targets nuclear factor-κB-repressing factor and claudin 1 to increase intestinal permeability. Gastroenterology. 2015;148: 158–169.e8. 25277410 10.1053/j.gastro.2014.09.037 PMC4303568

[19] Nourbakhsh M, Hauser H. Constitutive silencing of IFN-β promoter is mediated by NRF (NF-κB-repressing factor), a nuclear inhibitor of NF-κB. EMBO J. 1999;18: 6415–6425. 10562553 10.1093/emboj/18.22.6415 PMC1171704

[20] Yang M, Jia W, Wang D, Han F, Niu W, Zhang H, et al. Effects and Mechanism of Constitutive TL1A Expression on Intestinal Mucosal Barrier in DSS-Induced Colitis. Dig Dis Sci. 2019;64: 1844–1856. doi: 10.1007/s10620-019-05580-z 30949903

[21] Drossman DA, Hasler WL. Rome IV—Functional GI disorders: Disorders of gut-brain interaction. Gastroenterology. 2016;150: 1257–1261. doi: 10.1053/j.gastro.2016.03.035 27147121

[22] Zhu H, Xiao X, Chai Y, Li D, Yan X, Tang H. MiRNA-29a modulates visceral hyperalgesia in irritable bowel syndrome by targeting HTR7. Biochem Biophys Res Commun. 2019;511: 671–678. doi: 10.1016/j.bbrc.2019.02.126 30827505

[23] Al-Chaer ED, Kawasaki M, Pasricha PJ. A new model of chronic visceral hypersensitivity in adult rats induced by colon irritation during postnatal development. Gastroenterology. 2000;119: 1276–1285. doi: 10.1053/gast.2000.19576 11054385

[24] Liang J, Liang J, Hao H, Lin H, Wang P, Wu Y, et al. The extracts of Morinda officinalis and its hairy roots attenuate dextran sodium sulfate-induced chronic ulcerative colitis in mice by regulating inflammation and lymphocyte apoptosis. Front Immunol. 2017;8: 1–17. 28824631 10.3389/fimmu.2017.00905 PMC5539173

[25] Asseman C, Mauze S, Leach MW, Coffman RL, Powrie F. An essential role for interleukin 10 in the function of regulatory T cells that inhibit intestinal inflammation. J Exp Med. 1999;190: 995–1003. doi: 10.1084/jem.190.7.995 10510089 PMC2195650

[26] Wang Y, Liu J, Huang Z, Li Y, Liang Y, Luo C, et al. Coptisine ameliorates DSS-induced ulcerative colitis via improving intestinal barrier dysfunction and suppressing inflammatory response. Eur J Pharmacol. 2021;896. doi: 10.1016/j.ejphar.2021.173912 33508280

[27] Lobo B, Ramos L, Martínez C, Guilarte M, González-Castro AM, Alonso-Cotoner C, et al. Downregulation of mucosal mast cell activation and immune response in diarrhoea-irritable bowel syndrome by oral disodium cromoglycate: A pilot study. United Eur Gastroenterol J. 2017;5: 887–897. doi: 10.1177/2050640617691690 29026603 PMC5625876

[28] Wen ZS, Du M, Tang Z, Zhou TY, Zhang ZS, Song HH, et al. Low molecular seleno-aminopolysaccharides protect the intestinal mucosal barrier of rats under weaning stress. Int J Mol Sci. 2019;20. doi: 10.3390/ijms20225727 31731602 PMC6888692

[29] Lv M, Zhong Z, Huang M, Tian Q, Jiang R, Chen J. lncRNA H19 regulates epithelial–mesenchymal transition and metastasis of bladder cancer by miR-29b-3p as competing endogenous RNA. Biochim Biophys Acta—Mol Cell Res. 2017;1864: 1887–1899. doi: 10.1016/j.bbamcr.2017.08.001 28779971

[30] Dai Y, Mao Z, Han X, Xu Y, Xu L, Yin L, et al. MicroRNA-29b-3p reduces intestinal ischaemia/reperfusion injury via targeting of TNF receptor-associated factor 3. Br J Pharmacol. 2019;176: 3264–3278. doi: 10.1111/bph.14759 31167039 PMC6692574

[31] Scuderi SA, Casili G, Lanza M, Filippone A, Paterniti I, Esposito E, et al. Modulation of nlrp3 inflammasome attenuated inflammatory response associated to diarrhea-predominant irritable bowel syndrome. Biomedicines. 2020;8: 1–16. doi: 10.3390/biomedicines8110519 33233503 PMC7699594

[32] Demircan M, Cetin S, Uguralp S, Sezgin N, Karaman A, Gozukara EM. Plasma D-lactic acid level: A useful marker to distinguish perforated from acute simple appendicitis. Asian J Surg. 2004;27: 303–305. doi: 10.1016/S1015-9584(09)60056-7 15564184

[33] Miyoshi J, Miyamoto H, Goji T, Taniguchi T, Tomonari T, Sogabe M, et al. Serum diamine oxidase activity as a predictor of gastrointestinal toxicity and malnutrition due to anticancer drugs. J Gastroenterol Hepatol. 2015;30: 1582–1590. doi: 10.1111/jgh.13004 25968084

[34] Stevens BR, Goel R, Seungbum K, Richards EM, Holbert RC, Pepine CJ, et al. Altered Gut Microbiome in Anxiety or Depression. Gut. 2018;67: 1555–1557. 10.1136/gutjnl-2017-314759 PMC5851874 28814485

[35] Pinto-Sanchez MI, Ford AC, Avila CA, Verdu EF, Collins SM, Morgan D, et al. Anxiety and depression increase in a stepwise manner in parallel with multiple FGIDs and symptom severity and frequency. Am J Gastroenterol. 2015;110: 1038–1048. doi: 10.1038/ajg.2015.128 25964226

[36] Bradesi S, Schwetz I, Ennes HS, Lamy CMR, Ohning G, Fanselow M, et al. Repeated exposure to water avoidance stress in rats: A new model for sustained visceral hyperalgesia. Am J Physiol—Gastrointest Liver Physiol. 2005;289. doi: 10.1152/ajpgi.00500.2004 15746211

[37] Yan B, Guo Q, Fu FJ, Wang Z, Yin Z, Wei YB, et al. The role of miR-29b in cancer: Regulation, function, and signaling. Onco Targets Ther. 2015;8: 539–548. doi: 10.2147/OTT.S75899 25767398 PMC4354468

[38] Hu J, Zhu XH, Zhang XJ, Wang PX, Zhang R, Zhang P, et al. Targeting TRAF3 signaling protects against hepatic ischemia/reperfusions injury. J Hepatol. 2016;64: 146–159. doi: 10.1016/j.jhep.2015.08.021 26334576

[39] Sun Y, Zhou Y, Shi Y, Zhang Y, Liu K, Liang R, et al. Expression of miRNA-29 in Pancreatic β Cells Promotes Inflammation and Diabetes via TRAF3. Cell Rep. 2021;34: 108576. 33406428 10.1016/j.celrep.2020.108576

[40] Zhang B, Shetti D, Fan C, Wei K. miR-29b-3p promotes progression of MDA-MB-231 triple-negative breast cancer cells through downregulating TRAF3. Biol Res. 2019;52: 38. doi: 10.1186/s40659-019-0245-4 31349873 PMC6659300

[41] Profile C, E-mediated I, Irritable W, Syndrome B, Diarrhea W. Cytokine Profile and Immunoglobulin E-mediated Serological Food Hypersensitivity in Patients With Irritable Bowel Syndrome With Diarrhea. 2018;24: 415–421. 10.5056/jnm17114 PMC6034672 29739174

[42] Ã AW. Role of NF- k B activation in intestinal immune homeostasis. Int Jl Med Microbiol. 2010;300: 49–56. 10.1016/j.ijmm.2009.08.007 19781989

[43] Nighot M, Rawat M, Al-Sadi R, Castillo EF, Nighot P, Ma TY. Lipopolysaccharide-Induced Increase in Intestinal Permeability Is Mediated by TAK-1 Activation of IKK and MLCK/MYLK Gene. Am J Pathol. 2019;189: 797–812. doi: 10.1016/j.ajpath.2018.12.016 30711488 PMC6446229

[44] Ma TY, Boivin MA, Ye D, Pedram A, Said HM, Thomas Y, et al. Mechanism of TNF- α modulation of Caco-2 intestinal epithelial tight junction barrier: role of myosin light-chain kinase protein expression. 2005;0001: 422–430. 10.1152/ajpgi.00412.2004 15701621

[45] Marchiando AM, Shen L, Graham WV, Edelblum KL, Carrie A, Guan Y, et al. NIH Public Access. 2012;140: 1208–1218.

