# Supplementary material for: MicroRNA-29b-3p promotes intestinal permeability in IBS-D via targeting TRAF3 to regulate the NF-κB-MLCK signaling pathway
Source: PLoS One. 2023 Jul 10;18(7):e0287597. doi: 10.1371/journal.pone.0287597 (PMC10332595; doi:10.1371/journal.pone.0287597)
Supplement: S1 File — (DOCX) [file pone.0287597.s001.docx]

**Table 1** Histological score and serum D-LA levels in humans.

| Histological score | D-LA (mmol/L) |
| --- | --- |
| 1 | 1.69 |
| 2 | 1.96 |
| 1 | 1.37 |
| 1 | 1.68 |
| 1 | 1.79 |
| 1 | 1.82 |
| 2 | 7.28 |
| 3 | 8.53 |
| 2 | 7.38 |
| 2 | 6.45 |
| 2 | 5.74 |
| 3 | 8.68 |

**Table 2** Histological score and serum DAO levels in humans.

| Histological score | DAO (ng/mL) |
| --- | --- |
| 1 | 3.61 |
| 2 | 3.01 |
| 1 | 2.94 |
| 1 | 3.06 |
| 1 | 2.91 |
| 1 | 2.75 |
| 2 | 4.23 |
| 3 | 4.53 |
| 2 | 4.22 |
| 2 | 4.19 |
| 2 | 4.04 |
| 3 | 4.65 |

**Table 3** Histological score and serum LPS levels in humans.

| Histological score | LPS (EU/L) |
| --- | --- |
| 1 | 5.55 |
| 2 | 6.64 |
| 1 | 5.83 |
| 1 | 5.69 |
| 1 | 6.54 |
| 1 | 5.79 |
| 2 | 7.83 |
| 3 | 8.26 |
| 2 | 6.42 |
| 2 | 7.44 |
| 2 | 7.97 |
| 3 | 8.18 |

**Figure 1**





Figure 1 Correlation analysis of serum D-LA levels and histological score

in humans

**Figure 2**





Figure 2 Correlation analysis of serum DAO levels and histological score

in humans

**Figure 3**





Figure 3 Correlation analysis of serum D-LA levels and histological score

in humans
