# Supplementary material for: MicroRNA-29b-3p promotes intestinal permeability in IBS-D via targeting TRAF3 to regulate the NF-κB-MLCK signaling pathway
Source: PLoS One. 2023 Jul 10;18(7):e0287597. doi: 10.1371/journal.pone.0287597 (PMC10332595; doi:10.1371/journal.pone.0287597)
Supplement: S1 Raw images — (PDF) [file pone.0287597.s002.pdf]

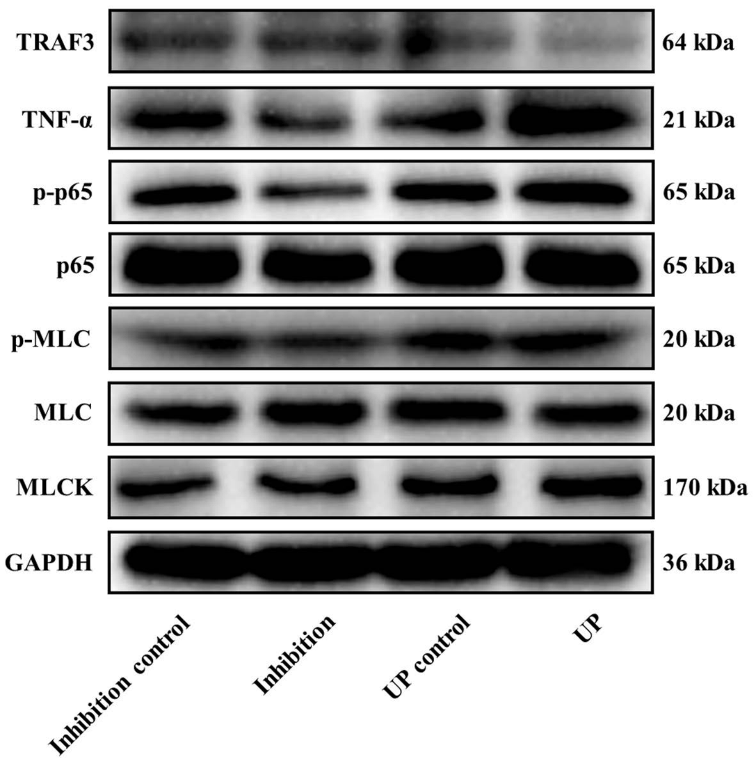
TRAF3
TNF-α
p-p65
p65
p-MLC
MLC
MLCK
GAPDH
64 kDa
21 kDa
65 kDa
65 kDa
20 kDa
20 kDa
170 kDa
36 kDa
Inhibition control
Inhibition
UP control
UP

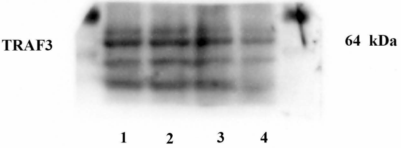


1 Inhibition control
2 Inhibition
3 UP control
4 UP

1 Inhibition control
2 Inhibition
3 UP control
4 UP

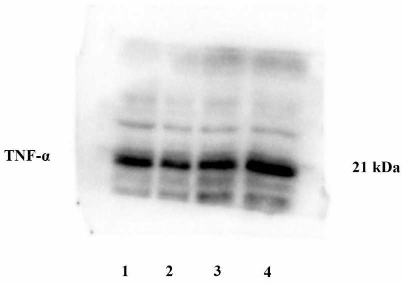

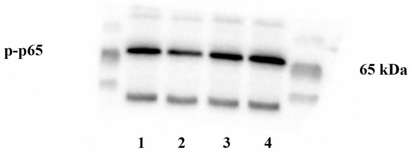


1 Inhibition control
2 Inhibition
3 UP control
4 UP

p65

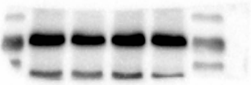

65 kDa

1 2 3 4

1 Inhibition control
2 Inhibition
3 UP control
4 UP

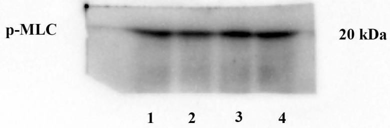


1 Inhibition control
2 Inhibition
3 UP control
4 UP

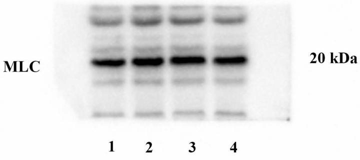


1 Inhibition control
2 Inhibition
3 UP control
4 UP

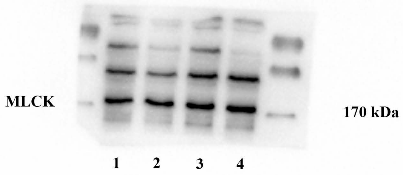


1 Inhibition control
2 Inhibition
3 UP control
4 UP

1 Inhibition control
2 Inhibition
3 UP control
4 UP

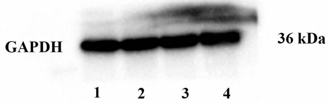

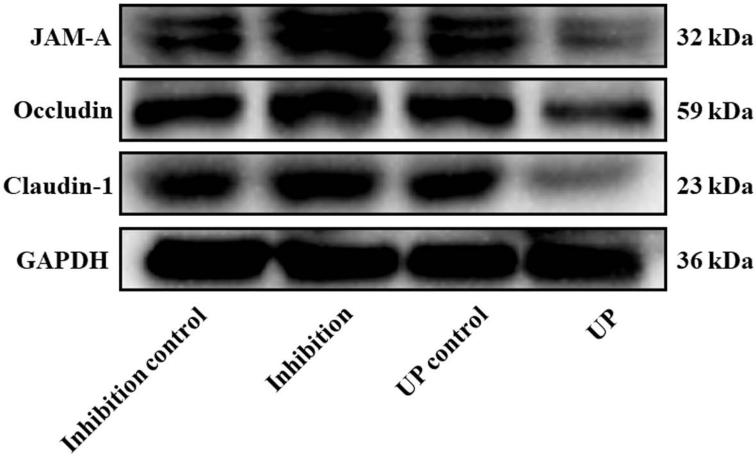
JAM-A
Occludin
Claudin-1
GAPDH
32 kDa
59 kDa
23 kDa
36 kDa
Inhibition control
Inhibition
UP control
UP

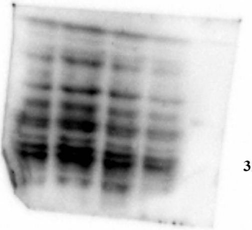

JAM-A

32 kDa

1 2 3 4

1 Inhibition control
2 Inhibition
3 UP control
4 UP

1 Inhibition control
2 Inhibition
3 UP control
4 UP

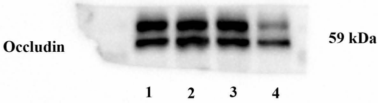

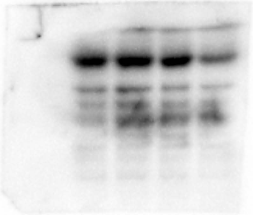


1 Inhibition control
2 Inhibition
3 UP control
4 UP

1 Inhibition control
2 Inhibition
3 UP control
4 UP

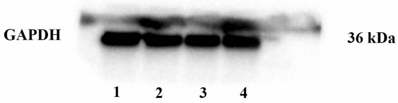

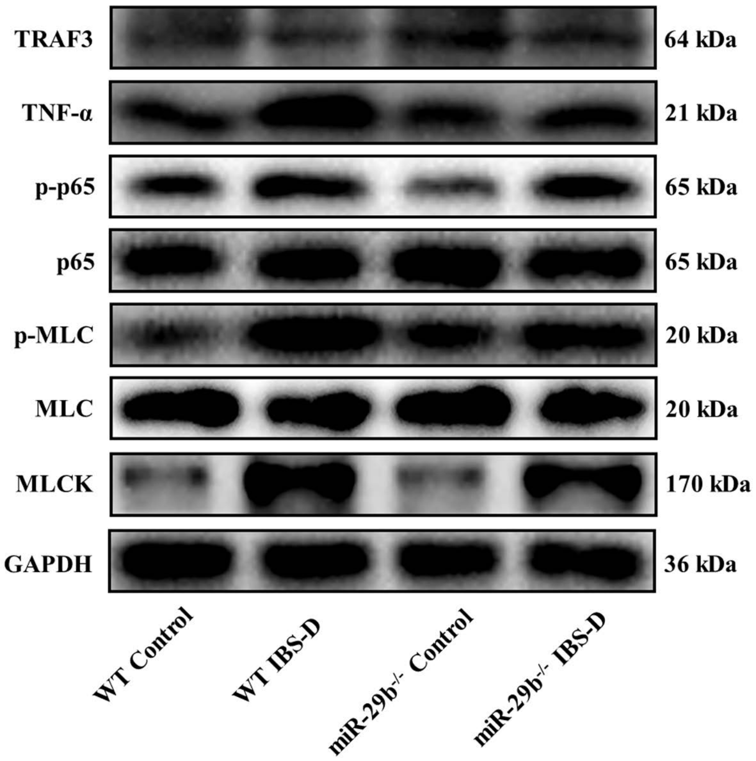

TRAF3
64 kDa
TNF-α
21 kDa
p-p65
65 kDa
p65
65 kDa
p-MLC
20 kDa
MLC
20 kDa
MLCK
170 kDa
GAPDH
36 kDa
WT Control
WT IBS-D
miR-29b-/- Control
miR-29b-/- IBS-D

1 WT Control
2 WT IBS-D
3 miR-29-/- Control
4 miR-29-/- IBS-D

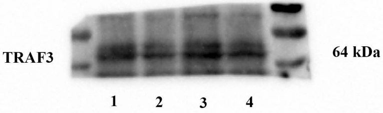

1 WT Control
2 WT IBS-D
3 miR-29-/- Control
4 miR-29-/- IBS-D

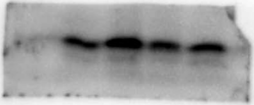

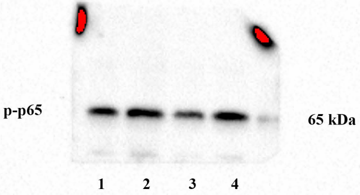


1 WT Control
2 WT IBS-D
3 miR-29-/- Control
4 miR-29-/- IBS-D

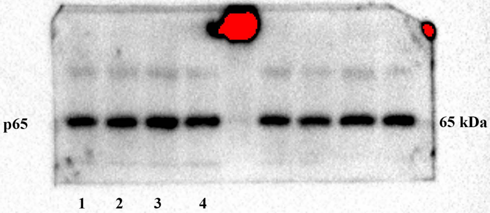


1 WT Control
2 WT IBS-D
3 miR-29-/- Control
4 miR-29-/- IBS-D

1 WT Control
2 WT IBS-D
3 miR-29-/- Control
4 miR-29-/- IBS-D

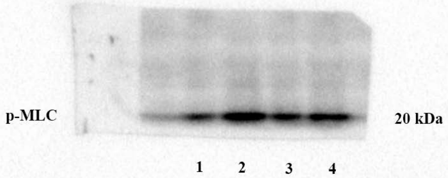

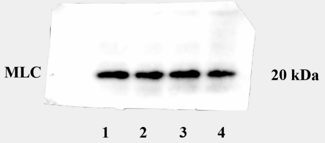


1 WT Control
2 WT IBS-D
3 miR-29-/- Control
4 miR-29-/- IBS-D

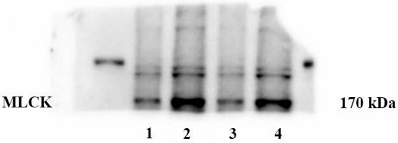


1 WT Control
2 WT IBS-D
3 miR-29-/- Control
4 miR-29-/- IBS-D

1 WT Control
2 WT IBS-D
3 miR-29-/- Control
4 miR-29-/- IBS-D

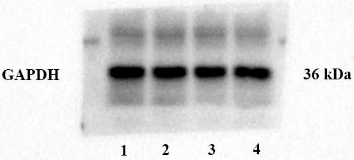

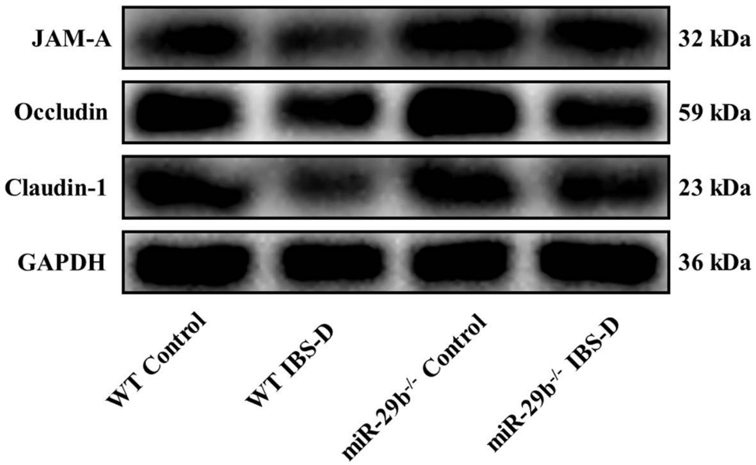
JAM-A
Occludin
Claudin-1
GAPDH
32 kDa
59 kDa
23 kDa
36 kDa
WT Control
WT IBS-D
miR-29b-/- Control
miR-29b-/- IBS-D

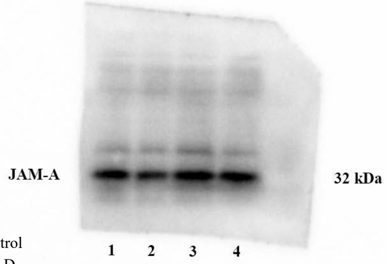


1 WT Control
2 WT IBS-D
3 miR-29-/- Control
4 miR-29-/- IBS-D

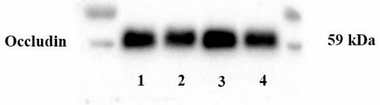


1 WT Control
2 WT IBS-D
3 miR-29-/- Control
4 miR-29-/- IBS-D

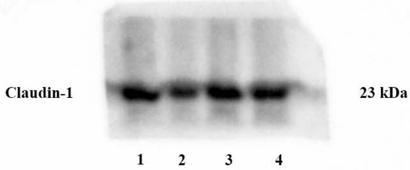


1 WT Control
2 WT IBS-D
3 miR-29-/- Control
4 miR-29-/- IBS-D

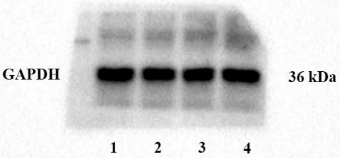


1 WT Control
2 WT IBS-D
3 miR-29-/- Control
4 miR-29-/- IBS-D
